# Endorsement of artificial intelligence guidelines across leading endocrinology journals: a cross-sectional analysis

**DOI:** 10.3389/fendo.2026.1767254

**Published:** 2026-04-14

**Authors:** Eva Chen, Amy Nguyen, Kellen Keefer, Eddy Bagaruka, Mahad Chaudhry, Annes Elfar, Patrick Crotty, Alec Young, Andrew V. Tran, Alicia Ito Ford, Matt Vassar

**Affiliations:** 1 Office of Medical Student Research, Oklahoma State University Center for Health Sciences, Tulsa, OK, United States; 2 Department of Psychiatry and Behavioral Sciences, Oklahoma State University Center for Health Sciences, Tulsa, OK, United States

**Keywords:** artificial intelligence, journal policy, reporting guidelines, reproducibility, transparency

## Abstract

**Background:**

With expanding applications of artificial intelligence (AI) within the research pipeline of endocrinology, it is essential that journals uphold explicit AI usage policies that maintain the rigor and integrity of published research. In this review, we aim to evaluate current AI policies of leading endocrinology journals to assess the current landscape of research and the implications of its progression.

**Methods:**

We conducted a cross-sectional review of the top endocrinology journals using the SCImago Journal Ranking (SJR) database. From November 2024 to July 2025, we reviewed AI usage guidelines from publicly available Instructions for Authors, including authorship, manuscript writing, and content/image generation. We also assessed whether journals endorsed AI-specific reporting guidelines (e.g., CONSORT-AI, SPIRIT-AI). Data were extracted independently and in duplicate using a standardized form. Reproducibility was supported through protocol registration on Open Science Framework.

**Results:**

Of the top 100 endocrinology journals, 84.0% (84/100) mentioned AI in their Instructions for Authors and 79.0% (79/100) required disclosure of AI use during submission. Although no journals (0/100) permitted AI tools for authorship, 64.0% (64/100) allowed its use in manuscript writing, 22.0% (22/100) for content generation, and 50.0% (50/100) for image generation. Despite these guidelines, only one (1.0%; 1/100) journal required a specific reporting guideline, and very few endorsed AI statements by the IMCJE (9/100), COPE (12/100), or WAME (0/100). No statistically significant correlations were identified between AI usage policies and SJR or impact factor.

**Conclusion:**

Many leading endocrinology journals have addressed AI use; however, their policies remain incomprehensive. It is critical that publishers and their journals establish explicit guidelines regarding the use of AI tools to promote transparent, reproducible, and reliable research.

## 1 Introduction

The healthcare field is currently undergoing rapid transformation with the integration of artificial intelligence (AI) into clinical practice, and endocrinology is no exception. Advancements in AI have contributed to medical practice by enhancing diagnostic accuracy, streamlining patient management, and enabling personalized treatment approaches. In endocrinology specifically, AI has been implemented in patient-centered algorithms for the use of hybrid closed loop automated insulin delivery in diabetes treatment, effectively improving glycemic control and decreasing risk of severe hypoglycemia (1). The potential of AI in endocrinological care continues to magnify with strong developments observed in trials for individualized treatment systems, thyroid nodule diagnosis, adrenal imaging interpretation, among many more (2–5). While AI offers promising opportunities in endocrinology application and research, its quick emergence introduces potential challenges that compromise the quality and reliability of scientific advancements.

An inherent weakness of AI algorithms is their dependence on the quality and quantity of the data they are trained on (6). Clinical datasets are frequently incomplete, different, or inconsistently documented, which can introduce bias into algorithm development. Thus, models trained on such data may produce inaccurate predictions or demonstrate limited generalizability, ultimately compromising patient safety and outcomes (6, 7). Furthermore, the “black-box” nature of some AI models obscures the decision-making process regarding how AI models reach a conclusion. Such lack of transparency hinders acceptance among medical professionals who require proper interpretability before integrating AI in clinical decision making (6, 8, 9). The lack of clarity raises concerns about reproducibility and legitimacy of AI-driven research. Ethical considerations further complicate the adoption of AI in endocrinology. Issues such as data privacy, informed consent, and the potential misuse of AI-generated findings demand robust oversight and regulatory frameworks (10). Addressing these challenges is essential to ensure the responsible and ethical application of AI in healthcare.

Organizations like the International Committee on Medical Journal Editors (ICMJE), Committee on Publication Ethics (COPE), and World Association of Medical Editors (WAME) have introduced AI-specific guidelines to promote transparency across scientific literature involving AI systems (11–14). Reporting guidelines such as CONSORT-AI and SPIRIT-AI aim to verify completeness in clinical trials that implement AI systems (15, 16). Furthermore, journal-specific generative AI policies address topics of scholarly integrity, authorship authority, and ethical transparency in works that used AI tools. Limited research exists evaluating whether endocrinology journals have adopted AI-specific reporting guidelines or implemented generative AI policies. Such a gap highlights the urgent need to critically assess and address publication standards in the field. Given the growing role of AI across both research development and scientific communication, it is important to understand how journals address these issues within their editorial policies. Considering these developments, our study aims to investigate the presence and content of AI-related policies across leading endocrinology journals, focusing primarily on guidance related to the use of generative AI in manuscript preparation while also documenting references to AI-specific reporting guidelines.

## 2 Methods

### 2.1 Study design

We conducted a cross-sectional review of the manuscript submission guidelines of the top 100 journals ranked by the 2023 SCImago Journal Ranking (SJR). Data was collected from the Instructions For Authors’ page for each journal. For this study, we adhered to the Strengthening the Reporting of Observational Studies in Epidemiology (STROBE) guidelines. Ethical approval for this study was obtained from the Institutional Review Board of Oklahoma State University Center for Health Sciences (Study #2024145).

### 2.2 Search strategy

Eligible journals were identified by consultation between two investigators (PC, AVT) and the medical research librarian. The 2023 SJR was used to obtain 100 journal listings to capture a representative sample of leading journals while maintaining a dataset that was feasible for systematic manual evaluation. SCImago is an online platform that analyzes metrics, such as h-index and SCImago Journal Rank Indicator to rank journals (17). The SJR offers a more comprehensive evaluation of a publication’s influence and reach (18). The rankings were made annually through Elsevier’s subscription-based Scopus database, giving us a large sample of scientific journals for the comprehensive analysis of this study. Within the 2023 SCImago journal listings included in this study, each peer-reviewed journal was identified under the “Medicine” subject area and “Endocrinology, Diabetes, and Metabolism” subject category. Furthermore, the “Journal” filter was applied to ensure that only clinical journals were included; book series, conferences, proceedings, and trade journals were excluded.

### 2.3 Inclusion and exclusion criteria

The top 100 peer-reviewed journals in the “Endocrinology, Diabetes, and Metabolism” subject category were analyzed. Journals were included if journal websites provide “Instructions for Authors” pages in English and had to be actively publishing research in the fields of endocrinology, diabetes, or metabolism. In contrast, journals were excluded from our analysis if they met any of the following criteria: (1) were discontinued, (2) lacked contact information for the editorial office on their website (to minimize bias by allowing editors the opportunity to clarify their publication policies), (3) were published in non-English and did not offer a translation option, or (4) were not related to endocrinology, diabetes, or metabolism.

### 2.4 Data extraction process

Two investigators (EC, AN) independently extracted data from the “Instructions for Authors” pages of all included journals in a masked, duplicated manner. The data extracted by the investigators includes authorship criteria, publishing policies, and any editorials or updates from journals and publishing companies regarding the use of AI, large language models, and chatbots. The data were collected using a standardized pilot-tested Google Form designed *a priori* by investigators PC and AVT. After the authors completed their independent extractions, the two investigators (EC, AN) reconciled their data. If at any point the investigators could not come to a consensus with the data, a third investigator (PC) would resolve the discrepancy. Following completion of the data extraction period, the URL for each journal’s “Instructions for Authors” page was searched using the Internet Archive’s Wayback Machine (19). Archived snapshots within the data extraction timeframe were reviewed to identify any changes to the “Instructions for Authors” pages. If changes were detected, they were examined to determine whether they involved policies related to the use of artificial intelligence. When AI-related policy changes were identified, the journal’s “Instructions for Authors” were re-extracted to ensure the most accurate and up-to-date information corresponding to the data extraction timeframe was included.

### 2.5 Data items

The following data were extracted for each journal included in the study: title, geographic region, quartile within category, SJR score, 2023 journal impact factor, publishing company, and acknowledgment of ICMJE, COPE, and WAME. Publishing companies were standardized to address variations in naming conventions to maintain consistency. For example, journals listed under different variations of a parent company’s name, such as “Elsevier B.V.” and “Elsevier Ltd.,” were uniformly categorized as “Elsevier.” Similarly, subsidiaries or imprints of larger publishing houses were consolidated under their parent company name where applicable.

We searched the following keywords to determine if the “Instructions for Authors” pages mentioned AI: “Artificial Intelligence,” “AI,” “ChatGPT,” “Large Language Models,” “Language Models,” “LLM.” Additionally, we assessed relevant guideline sections and any external links provided to ensure that our search was comprehensive and robust. Relevant guideline sections included journal webpages or policy documents describing rules or guidance related to AI regarding authorship, manuscript writing, content and image generation, and disclosure. During data extraction, investigators reviewed sections such as “Author Guidelines,” “Ethics Guidelines,” and any linked publisher or parent organization policies. If no relevant results were found, we classified the journals as not having a policy on AI use.

For journals that referenced AI in their “Instructions for Authors,” we extracted specific details regarding AI use, including its application for authorship, manuscript writing, content generation, and image generation. Specifically, we sought statements regarding AI-specific reporting guidelines, such as: (1) Consolidated Standards of Reporting Trials involving Artificial Intelligence (CONSORT-AI), (2) Standard Protocol Items: Recommendations for Interventional Trials involving Artificial Intelligence (SPIRIT-AI), (3) Fairness Universality Traceability Usability Robustness Explainability Artificial Intelligence solutions (FUTURE-AI), (4) Minimum Information about Clinical Artificial Intelligence Modeling (MI-CLAIM), (5) Minimum Information for Medical AI Reporting (MINIMAR), (6) CheckList for Artificial Intelligence in Medical imaging (CLAIM), (7) Must Artificial Intelligence Criteria-10 (MAIC-10), (8) Radiomics Quality Score (RQS), (9) Image Biomarker Standardization Initiative (IBSI), (10) CheckList for EvaluAtion of Radiomics research (CLEAR), (11) Consolidated Health Economic Evaluation Reporting Standards for Interventions That Use Artificial Intelligence (CHEERS-AI). While our study included a comprehensive set of AI-related reporting guidelines, it did not encompass all existing AI reporting guidelines. Therefore, if a journal referenced additional AI reporting guidelines not listed above, we documented them accordingly. We recognize that not all AI reporting guidelines are directly applicable to the fields of endocrinology, diabetes, or metabolism. For example, in those listed above, CLAIM, RQS, IBSI, and CLEAR are geared more towards the field of radiology; however, they are included here for completeness since imaging of certain pathologies like thyroid nodules and pituitary tumors are within the endocrinology realm.

### 2.6 Editorial outreach

Journals that did not include AI usage statements on their “Instructions for Author” page were contacted via standardized emails to the Editor-in-Chief or a member of the editorial office to inquire about AI policies. To improve response rates, emails were sent once a week for three weeks (20). If an email response was not received a week after the last attempt, it was assumed that the “Instructions for Authors” were up-to-date and that editors were not actively updating their policies. All responses from journals and editors were documented, and a copy of the standardized email was uploaded to the Open Science Framework (OSF) for transparency (21).

### 2.7 Outcomes

The primary endpoint of this study was to assess how AI-related editorial policies (i.e. authorship, manuscript writing, image generation, and disclosure requirements) are addressed in journal “Instructions for Authors” pages. The secondary endpoint focused on evaluating the endorsement of AI-specific reporting guidelines. By systematically analyzing these aspects, our study aimed to identify existing policies, highlight gaps in guidance, and assess the readiness of Endocrinology journals to adapt to AI’s evolving role in research and publication.

### 2.8 Data synthesis

Descriptive statistics were generated to summarize journal AI policies, focusing on four key areas: (1) AI-generated content, (2) AI-generated images, (3) AI authorship inclusion, and (4) AI-assisted manuscript writing. Bias analysis was not included, as the data were from a direct evaluation of the “Instructions for Authors” pages rather than an assessment of individual studies. Correlational analyses were performed using R (version 4.2.1) and RStudio to evaluate the relationship between AI-related policies and journal characteristics, including rank, SJR score, publishing country, and impact factor.

To investigate the predictors of whether journals mentioned AI in their “Instructions for Authors,” a logistic regression analysis was performed. The analysis determined if the following factors correlated with journals mentioning AI: 2023 Impact Factor, SCImago Ranking, and continent categorization. We assessed model fit using the Akaike Information Criterion (AIC) and checked for multicollinearity among predictors using the Variance Inflation Factor (VIF) (22, 23). These analyses were also conducted using R (version 4.2.1) and RStudio.

### 2.9 Reproducibility

To promote transparency and reproducibility, all raw data collected, analysis scripts, standardized emails, and extraction forms are publicly available via OSF (21). Additionally, our original protocol and any amendments were uploaded to OSF.

## 3 Results

**Figure 1:**
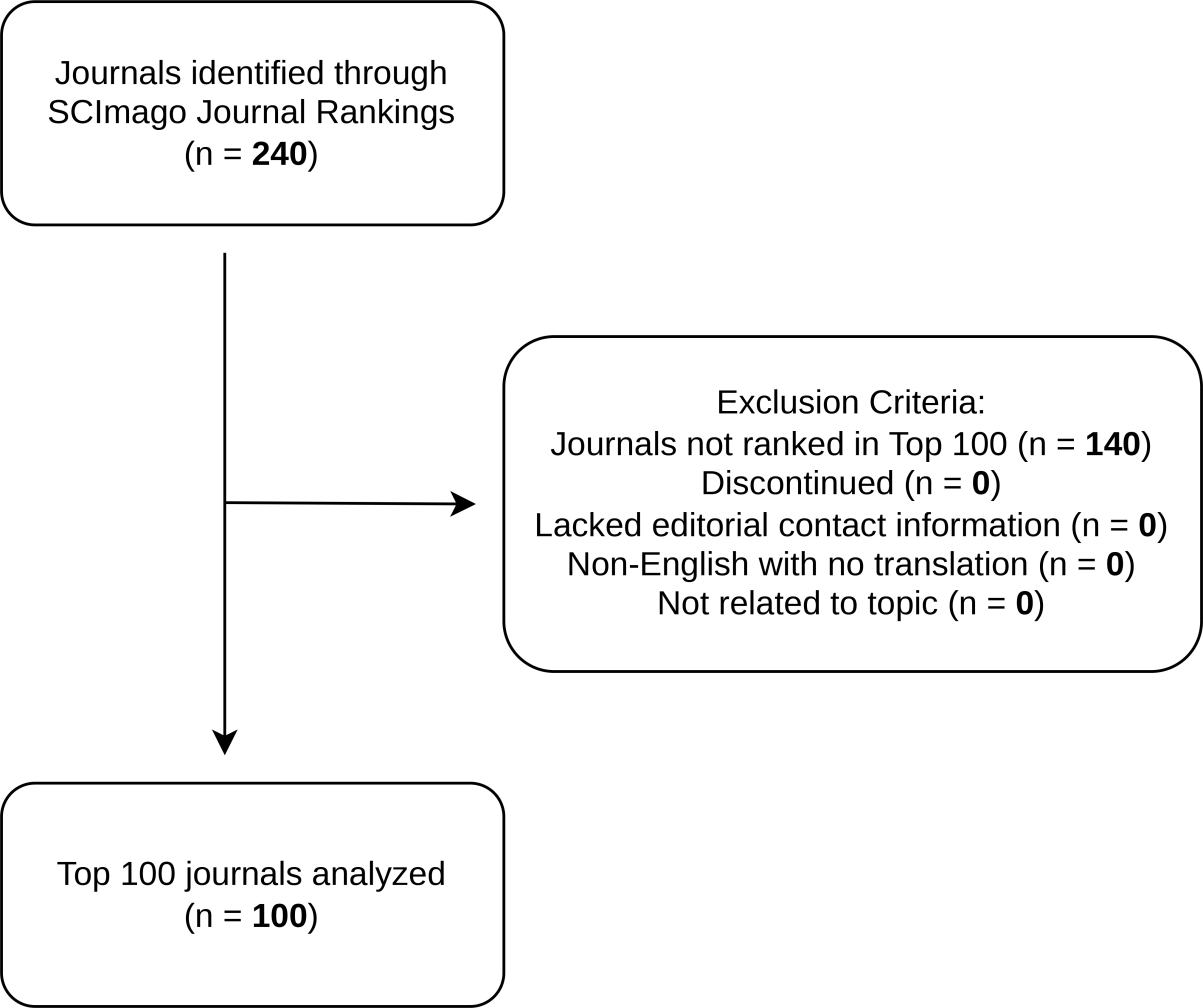
PRISMA Flowchart of study selection.

Using SCImago, our initial search identified 240 journals classified under Endocrinology, Diabetes, and Metabolism. For data extraction and analysis, we selected only the top 100 journals based on SJR, ensuring our analysis focused on the most reputable and influential journals in endocrinology research. See **Figure 1** for complete study selection.

The majority of the included journals were published in Europe (53/100) or North America (40/100), with the remaining journals published in Asia (4/100), Africa (2/100), and Australia (1/100). Nearly two-thirds of the journals were classified in the SJR Quartile 1 (61/100), with the remainder in Q2 (39/100). The 2023 Journal Impact Factor for the included journals ranged from 3.2 to 5.1 with a median impact factor of 3.8. Only one (1/100) journal did not list an impact factor. The most frequently encountered publishers in this study were Springer Nature (23/100), Elsevier (20/100), and Wiley Blackwell (16/100). See **Table 1** for a more detailed summary of journal characteristics.

**Table 1:** Journal characteristics.

Characteristic	N = 100
Continent, n (%)	
Europe	53 (53.0)
North America	40 (40.0)
Asia	4 (4.0)
Africa	2 (2.0)
Australia	1 (1.0)
SJR Quartile, n (%)	
Q1	61 (61.0)
Q2	39 (39.0)
SCImago Rank, Median (IQR)	51 (26 – 76)
2023 Journal Impact Factor, Median (IQR)	3.8 (3.2 – 5.1)
Unknown	1
Publisher, n (%)	
Springer Nature	23 (23.0)
Elsevier	20 (20.0)
Wiley Blackwell	16 (16.0)
American Diabetes Association	3 (3.0)
Bioscientifica	3 (3.0)
Endocrine Society	3 (3.0)
Mary Ann Liebert	3 (3.0)
Oxford University Press	3 (3.0)
Sage Publications	3 (3.0)
Frontiers Media	2 (2.0)
Hindawi	2 (2.0)
Karger	2 (2.0)
Lippincott Williams & Wilkins	2 (2.0)
W.B. Saunders	2 (2.0)
American Physiological Society	1 (1.0)
BMJ	1 (1.0)
Canadian Science Publishing	1 (1.0)
Cell Press	1 (1.0)
Humana Press	1 (1.0)
Korean Diabetes Association	1 (1.0)
Korean Endocrine Society	1 (1.0)
Korean Society for the Study of Obesity	1 (1.0)
Korean Society of Lipid and Atherosclerosis	1 (1.0)
Masson Editeur	1 (1.0)
MDPI	1 (1.0)
Taylor & Francis	1 (1.0)
Thieme Medical Publishers	1 (1.0)

Over three-fourths (84/100) of journals in this study mentioned AI in the Instructions for Authors page. However, few journals included ICMJE statements (9/100) and COPE statements (12/100), and none (0/100) included WAME statements. Additionally, only one (1/100) journal mentioned AI specific reporting guidelines and required adherence to said guidelines, particularly CONSORT-AI, whereas the remaining (99/100) did not specify their stances. See **Table 2** for further characteristics of journal guidelines.

**Table 2:** General journal guidelines.

Characteristic	N = 100
AI mentioned in the Instructions for Authors, n (%)	
Yes	84 (84.0)
No	16 (16.0)
ICMJE Statement, n (%)	
Yes	9 (9.0)
No	91 (91.0)
COPE Statement, n (%)	
Yes	12 (12.0)
No	88 (88.0)
WAME Statement, n (%)	
Yes	0 (0.0)
No	100 (100.0)
AI Specific Reporting Guideline, n (%)	
Yes	1 (1.0)
No	99 (99.0)
Journal recommend or require adherence to said guideline(s), n (%)	
NA	99 (99.0)
Required	1 (1.0)
Specific Guidelines, n (%)	
CONSORT-AI, SPIRIT-AI	1 (100.0)
Unknown	99

Analysis of journal AI guidelines revealed that 78.0% (78/100) of journals prohibited AI tools for authorship. No journals (0/100) permitted AI authorship, while the remaining journals (22/100) did not specify their stance on the matter. Authors are required to disclose the use of AI tools in 79.0% (79/100) of the journals upon manuscript submission. The remaining 21.0% (21/100) of journals did not provide any statements regarding disclosure of AI tool use. Although more than half of journals specified that they allowed use of AI tools in manuscript writing (64/100), the remaining journals (35/100) did not state their position on the matter, with only one journal (1/100) explicitly prohibiting the use of AI tools for manuscript writing. AI use for content generation was permitted for 22.0% (22/100) of the journals, while the remaining journals either prohibited (38/100) or did not provide statements on AI use in this context (40/100). Regarding image generation, however, exactly half (50/100) of the journals allowed AI use; five journals (5/100) prohibited AI image generation, while the remaining 45.0% (45/100) did not specify their policies on this issue. See **Table 3** for more information.

**Table 3:** Journal AI guidelines.

Characteristic	N = 100
AI tools allowed for Authorship, n (%)	
Yes	0 (0.0)
No	78 (78.0)
Not Stated	22 (22.0)
Require authors to disclose the use of AI during submission, n (%)	
Yes	79 (79.0)
No	0 (0.0)
Not Stated	21 (21.0)
AI tools allowed in Manuscript Writing, n (%)	
Yes	64 (64.0)
No	1 (1.0)
Not Stated	35 (35.0)
AI tools allowed in content generation, n (%)	
Yes	22 (22.0)
No	38 (38.0)
Not Stated	40 (40.0)
AI tools allowed in image generation, n (%)	
Yes	50 (50.0)
No	5 (5.0)
Not Stated	45 (45.0)

Of the journals that did mention AI use within their guidelines (84/100), none (0/84) permitted AI tools for authorship, whereas the other journals prohibited AI tools for authorship (78/84) or did not state their stance (6/84). A majority of journals (79/84) required authors to disclose any use of AI during submission and the remainder (5/84) did not state specific requirements. Over three quarters of the journals (64/84) allowed AI tools for manuscript writing; only one (1/84) prohibited AI tools for manuscript writing and the others (19/84) did not provide any specific policy. However, regarding the use of AI tools for content generation, about a quarter of journals (22/84) allowed the use, with most (38/84) prohibiting it, and the remainder (24/84) not specifying. Additionally, over half (50/84) of journals allowed AI tools for image generation; five (5/84) prohibited their use; and the remaining (29/84) did not state. See **Table 4** for information regarding characteristics of the AI guidelines mentioned.

**Table 4:** AI guidelines in journals mentioning AI.

Characteristic	N = 84
AI tools allowed for Authorship, n (%)	
Yes	0 (0.0)
No	78 (92.9)
Not Stated	6 (7.1)
Require authors to disclose the use of AI during submission, n (%)	
Yes	79 (94.0)
No	0 (0.0)
Not Stated	5 (6.0)
AI tools allowed in Manuscript Writing, n (%)	
Yes	64 (76.2)
No	1 (1.2)
Not Stated	19 (22.6)
AI tools allowed in content generation, n (%)	
Yes	22 (26.2)
No	38 (45.2)
Not Stated	24 (28.6)
AI tools allowed in image generation, n (%)	
Yes	50 (59.5)
No	5 (6.0)
Not Stated	29 (34.5)

### 3.1 Correlation analysis

We performed biserial correlation analyses to evaluate the relationship between maintaining AI-related policies and journal characteristics, particularly the SJR and journal impact factor. Policies regarding AI tools for authorship and for manuscript writing were found to have no correlation with SJR or impact factor. However, policies regarding AI tools for content generation, image generation, and usage disclosures had a positive correlation with both SJR and impact factor, though statistically not significant.

### 3.2 Logistic regression analysis, AIC, and VIF

The analysis revealed a statistically significant negative association between SCImago and journals mentioning AI in their “Instructions for Authors.” The impact factor shows a similar but nonsignificant negative trend with higher impact factors correlating with lower endorsement of AI in these journals. There were no meaningful trends among journals published in different continents. A reasonable model fit was confirmed with the AIC. Additionally, there were no issues of multicollinearity found for any of the variables. See **Supplementary Table 1** for the predictors analyzed in journals mentioning AI.

## 4 Discussion

While most journals’ “Instructions for Authors” pages acknowledged AI use, explicit adoption of AI-specific reporting guidelines was rare, with only one journal mandating their use. Overall, our findings suggest a broad acceptance of AI assistance in manuscript preparation, with the notable restriction against AI-generated content and a consistent expectation that authors disclose AI use during submission. In contrast, 20.0-45.0% of journals provided no guidance on the AI policy characteristics that we evaluated, leaving ambiguity regarding the extent to which AI use is permitted. Collectively, these results highlight the need for journals to move beyond basic disclosure statements and adopt comprehensive, standardized AI reporting guidelines.

Our results are consistent with the broader literature, further indicating poor endorsement of specific AI reporting guidelines within clinical research. A 2023 study by Zhong et al. reviewed AI reporting guidelines across 117 radiology journals and found that only two (1.7%) journals included AI reporting guidelines (24). Another study by Ganjavi et al. reviewed the top 100 scientific journals and found that 87.0% released generative AI guidelines and 56.0% of the journals cited COPE, but no specific AI reporting guidelines were mentioned by name (25). Our results similarly reflect these findings since approximately 80.0% of the journals mention the extent to which AI use is allowed, but only one journal expanded their policies beyond manuscript development and established specific AI reporting guidelines specific to trial interventions. These results suggest that although many journals have general rules for the use of AI in manuscript preparation, there is an overall lack of endorsed, specific, and standardized AI reporting guidelines that account for the entirety of a study. AI reporting guidelines such as CONSORT-AI and SPIRIT-AI are implemented with the intent of setting international standards to “ensure complete and transparent reporting and enable effective evaluation of clinical trial protocols and results involving AI interventions.” (26) These extensions have already demonstrated their value in clinical trials, ensuring that AI interventions are clearly defined, properly evaluated, and transparently reported (27). Without such reporting guidelines in place, authors may not accurately report the use of AI within their study, affecting the transparency and reproducibility of study results. Additionally, AI usage could be misused to generate biased analysis. Methods involving AI use might even be excluded from manuscripts, further hindering transparency, reproducibility, and validity of the research. A 2025 study by AlFayyad et al. found that among 25,000 manuscript submissions, less than 1.0% self-disclosed the use of AI intrinsic to their methodology, including data analysis and image processing; a total of 5.7% self-disclosed the use of AI for any reason, including writing assistance (28). Moreover, it is essential that any AI use in methodology is disclosed to account for the potential biases of AI models and data output that can affect the reproducibility of AI-powered clinical trials (29).

Our results bring us to the question of why AI reporting guidelines are so scarce within the literature. One potential reason for this issue is that AI use in research is growing rapidly with constantly updated versions of AI able to conduct more robust analysis (30). With such advancements, reporting guidelines may be continuously evolving which may take time and resources away from producing and publishing new research. AI reporting may possibly be of lower priority with respect to all other manuscript requirements dictated by each journal. Another potential reason is that the abundance of AI reporting guidelines makes journals reluctant to adhere to any specific set of standards. For example, a 2024 study by Kolbinger et al. included 26 AI reporting guidelines alone (31). Perhaps with this many specific reporting guidelines, publishers may have difficulty deciding which ones to endorse, so they may allow for each journal’s discretion on whether to include or omit specific reporting guidelines. Our results show that journals from Springer Nature, Elsevier, and Wiley Blackwell account for 59.0% (59/100) of journals in our sample. Review of each of the top three publishers’ AI policies reveals that there is no requirement of a specific AI reporting guideline (such as CONSORT-AT or SPIRIT-AI), suggesting these entities allow for journal discretion regarding the implementation of specific AI reporting policies (32–34).

Furthermore, there is no single consensus regarding a specific practice across these three publishers, namely image generation. Our results found that nearly 60.0% (50/84) of journals stated they allow AI use for image generation, but with varying caveats. Springer Nature permits the publication of generative AI images if they are derived from agencies in which they maintain contractual relationships, are directly related to AI research, or can be reproduced via specific data sets (35). In a similar fashion, Elsevier only permits generative AI images if they are intrinsic to research design, whereas Wiley-Blackwell maintains a broad yet elaborate list of acceptable uses that may be difficult to discern (36, 37). Despite some circumstantial permissions among publishers, the wide variation between journals could indicate a higher level of laxity implementing generative AI policies, or even a widespread lack of adherence to established policy.

Our findings along with current literature highlight the necessity of standardizing the use of generative AI policies and AI reporting guidelines across all journals (31). A survey of Editors-in-Chief found that while nearly half of journals already use AI tools for plagiarism detection and data verification, editors remain cautious about using AI for scientific evaluation, emphasizing the need for clearer ethical and regulatory guidance (38). Journals at a minimum should provide specific statements regarding permissible AI uses to prevent any confusion or discrepancies in future publications. Such statements should include guidelines that regulate AI involvement in manuscript writing; clear permissions for generative uses for content and images; and accordance with AI statements of editorial bodies such as ICMJE, COPE, or WAME. Although a majority of the journals included in our review required disclosure of AI use during submission, it is critical that such disclosures become a standard among all journals given the pervasive nature of AI in scientific writing. Additionally, enforcement of specific AI reporting guidelines could help reduce the discrepancies in content and image generation rules across journals. Publishers such as Springer Nature, Elsevier, Wiley-Blackwell, and others should mandate specific AI reporting guidelines for all journals, therefore, removing journal discretion and potential noncompliance or ambiguity of AI use.

### 4.1 Strengths and limitations

Our study contains several strengths in its design. First, data extraction was conducted in a masked, duplicate manner to enhance accuracy. Second, we used a protocol developed *a priori* in order to provide a structured approach. Finally, two trained individuals performed the data extraction to ensure consistency. In contrast, we are aware that human error is possible; however, we sought to mitigate this limitation through reconciliation and discussion. Moreover, the “Instructions for Authors” pages among endocrinology journals were not standardized, and information may have been missed if an external link to further instructions was overlooked. However, we addressed this potential issue through similar reconciliation and discussion efforts. We also emailed editorial teams and used “Wayback Machine” to further mitigate this issue. Furthermore, we acknowledge our study’s limited AI-related search terms and suggest that future studies may benefit from a broader range of AI-related terminology as highlighted in González-Rivas et al. (39).

### 4.2 Conclusion

Our study demonstrates that while most leading endocrinology journals acknowledge the role of AI and require disclosure of its use, very few have adopted specific AI reporting guidelines. This lack of standardized policies introduces variability in how AI use is reported, creating uncertainty for authors and readers and ultimately limiting transparency and reproducibility in research. As AI continues to expand its role in endocrinology, from clinical care to research dissemination, clear and consistent editorial policies will be essential to safeguard scientific integrity. Journals should not only require disclosure of AI use but also mandate adherence to established reporting guidelines such as CONSORT-AI and SPIRIT-AI to ensure transparency across the entirety of a study through publication. Standardizing these requirements across publishers would help ensure responsible integration of AI into the scientific process, reduce ambiguity in publication practices, and foster greater trust in AI-driven research.

## Data Availability

The datasets presented in this study can be found in online repositories. The names of the repository/repositories and accession number(s) can be found below: https://osf.io/dc23r/?view_only=cc0f111783d7489090498a4982584251.
