# Supplementary material for: Endorsement of artificial intelligence guidelines across leading endocrinology journals: a cross-sectional analysis
Source: Front Endocrinol (Lausanne). 2026 Apr 14;17:1767254. doi: 10.3389/fendo.2026.1767254 (PMC13121116; doi:10.3389/fendo.2026.1767254)
Supplement: Supplementary file 1 [file Table1.docx]

**Supplemental Table 1.** Predictors of Journals Mentioning AI.

| **Predictors** | **OR** **(95% CI)** | ***p*-value** |
| --- | --- | --- |
| 2023 Journal Impact Factor | 0.91 (0.81 to 1.02) | 0.074 |
| SCImago Rank | 0.97 (0.94 to 0.99) | 0.017 |
| Continent |  |  |
| Africa | Not interpretable | > 0.99 |
| Asia | Not interpretable | > 0.99 |
| Australia | Not interpretable | > 0.99 |
| Europe | Not interpretable | > 0.99 |
| North America | Not interpretable | > 0.99 |
| OR = Odds Ratio, CI = Confidence Interval | | |
